# Cuproptosis-related lncRNA predict prognosis and immune response of lung adenocarcinoma

**DOI:** 10.1186/s12957-022-02727-7

**Published:** 2022-09-01

**Authors:** Fangwei Wang, Hongsheng Lin, Qisheng Su, Chaoqian Li

**Affiliations:** 1 Department of Respiratory Medicine, The First Affiliated Hospital of Guangxi Medical University, Nan’ning, China; 2 Department of Microbiology, School of Basic Medical Sciences, Guangxi Medical University, Nan’ning, China; 3 Department of Clinical Laboratory, The First Affiliated Hospital of Guangxi Medical University, Nan’ning, China

**Keywords:** Cuproptosis, lncRNAs, Signature, Prognosis, Immune, Drug sensitivity

## Abstract

**Background:**

Lung adenocarcinoma (LUAD) accounts for 50% of lung cancers, with high mortality and poor prognosis. Long non-coding RNA (lncRNA) plays a vital role in the progression of tumors. Cuproptosis is a newly discovered form of cell death that is highly investigated. Therefore, in the present study, we aimed to investigate the role of cuproptosis-related lncRNA signature in clinical prognosis prediction and immunotherapy and the relationship with drug sensitivity.

**Material and methods:**

Genomic and clinical data were obtained from The Cancer Genome Atlas (TCGA) and Gene Expression Omnibus (GEO) databases, and cuproptosis-related genes were obtained from cuproptosis-related studies. The prognostic signature was constructed by co-expression analysis and Cox regression analysis. Patients were divided into high and low risk groups, and then, a further series of model validations were carried out to assess the prognostic value of the signature. Subsequently, lncRNAs were analyzed for gene ontology (GO), Kyoto Encyclopedia of Genes and Genomes Enrichment (KEGG), immune-related functions, and tumor mutation burden (TMB). Finally, we used tumor immune dysfunction and exclusion (TIDE) algorithms on immune escape and immunotherapy of cuproptosis-related lncRNAs, thereby identifying its sensitivity toward potential drugs for LUAD.

**Results:**

A total of 16 cuproptosis-related lncRNAs were obtained, and a prognostic signature was developed. We found that high-risk patients had worse overall survival (OS) and progression-free survival (PFS) and higher mortality. Independent prognostic analyses, ROC, C-index, and nomogram showed that the cuproptosis-related lncRNAs can accurately predict the prognosis of patients. The nomogram and heatmap showed a distinct distribution of the high- and low-risk cuproptosis-related lncRNAs. Enrichment analysis showed that the biological functions of lncRNAs are associated with tumor development. We also found that immune-related functions, such as antiviral activity, were suppressed in high-risk patients who had mutations in oncogenes. OS was poorer in patients with high TMB. TIDE algorithms showed that high-risk patients have a greater potential for immune escape and less effective immunotherapy.

**Conclusion:**

To conclude, the 16 cuproptosis-related lncRNAs can accurately predict the prognosis of patients with LUAD and may provide new insights into clinical applications and immunotherapy.

## Background

Lung adenocarcinoma (LUAD) is a common type of lung cancer. It has one of the highest rates of incidence and mortality globally, posing a serious risk to human health [1]. Treatment options for lung cancer include surgery, radiotherapy, and chemotherapy [2]. Presently, LUAD is not diagnosed easily, and most patients are already at an advanced stage during diagnosis. This reduces the scope of surgery, and the distant spread of cancer cells causes serious health damage. Therefore, the treatment of LUAD should be improved through a rational treatment plan. Nowadays, tumor risk score prediction signatures are a non-invasive technique to identify patient survival. They can help to accurately predict prognosis and are gradually being used in clinical applications [3]. Therefore, prognostic signatures should be developed urgently to predict the long-term survival of patients with LUAD.

Copper is essential to all living organisms and plays a dual, paradoxical role in the cell, which helps to maintain intracellular copper concentrations at very low levels via a homeostatic mechanism [4]. Copper-induced cell death is mediated by iron-sulfur protein, when copper binds to acylated lipid products in the TCA cycle, leading to lipid acylated protein aggregation and loss of iron-sulfur cluster proteins. This results in proteotoxic stress and ultimately cell death, and this unique form of cell death is called cuproptosis [5]. Previous animal models and clinical trials have shown that copper deficiency inhibits tumor angiogenesis and growth [6]. The imbalance in copper levels can lead to uncontrolled tumor growth [7]. However, the exact mechanism underlying cuproptosis is still not completely elucidated. In hematopoietic cancers, a copper-dependent antitumor agent (IC25 (10)) exerts potent antitumor effects in vivo and in vitro [8]. In breast cancer, the endoplasmic reticulum-targeting copper (II) complex promotes phagocytosis of cancer cells by macrophages [9]. In pancreatic cancer, copper transporter 1 (SLC31A1) and copper chelator tetrathiomolybdate (TM) can promote autophagy and inhibit growth of cancer cells by reducing copper uptake [6]. Therefore, investigating the role of cuproptosis in cancer has great potential for clinical application.

Long non-coding RNAs (lncRNAs) are a class of RNA molecules with transcripts longer than 200 nt. They do not encode proteins but regulate gene expression at multiple epigenetic, transcriptional, and post-transcriptional levels in the form of RNA [10]. Previous in-depth studies on lncRNAs have reported that lncRNAs play a role in important pathologies and physiologies such as autophagy, development, differentiation, apoptosis, and cell cycle [11]. In tumor research, lncRNAs are differentially expressed in the lung, stomach, liver, colon, breast, and pancreatic cancers and play a role in tumor cell proliferation, migration, and invasion [11–15]. Many studies have reported that in colorectal cancer, lncRNA SH3PXD2A-AS1 expression is upregulated and promotes the growth of cancer cells, potentially serving as a diagnostic and therapeutic target [16]. DDX11-AS1 is a prognostic lncRNA with therapeutic potential in hepatocellular carcinoma by analyzing RNA-seq data [17]. In pancreatic cancer, lncRNA reprogramming (lncRNA-ROR) acts as a typical lncRNA with antitumor effects in vivo and in vitro. lncRNA-ROR can also inhibit cancer cell growth by activating miR-145 [15]. Notably, in endometrial cancer, autophagy-related lncRNAs can predict a patient’s prognosis. However, the role of lncRNAs in LUAD still needs to be further studied.

Cuproptosis-related lncRNAs can have clinical diagnostic and therapeutic implications for LUAD. In the present study, we used bioinformatics analysis to obtain cuproptosis-related lncRNAs and to analyze their biological functions and role in predicting the prognosis of patients with LUAD.

## Material and methods

### Data processing and identification of cuproptosis-related lncRNAs

RNA-Seq data of gene expression and the corresponding clinical and mutation data of LUAD were downloaded from the cancer genome atlas (TCGA) database (https://portal.gdc.cancer.gov/), which contained information on a total of 54 normal individuals and 501 patients with LUAD. Three Gene Expression Omnibus (GEO) datasets GSE30219 (*n*=307), GSE31210 (*n*=246), and GSE37745 (*n*=196) were downloaded from the GEO website (https://www.ncbi.nlm.nih.gov/geo/). RNA-Seq data were obtained according to the gene annotations from TCGA to distinguish lncRNAs. To identify potential cuproptosis-related lncRNAs, we performed a co-expression correlation analysis of lncRNA and cuproptosis-related gene expression profiles using the limma package at |R| >0.4 and *P* < 0.001.

### Construction of the prognostic cuproptosis-related lncRNA signature

A list of the cuproptosis-related genes was obtained from cuproptosis-related studies [18–22]. TCGA-LUAD data were randomly divided into training and testing groups in a 1:1 ratio. Univariate Cox analysis was performed in the training group, and Lasso Cox regression was performed on significantly expressed lncRNAs based on a 1000 ten-fold cross-validation to identify cuproptosis-related lncRNAs. Optimal prognostic lncRNAs were identified based on multivariate Cox regression analysis (*P* < 0.05), and the best model parameters were used for signature construction, followed by calculation of risk scores. Risk score = Exp lncRNA1 × β lncRNA1 + Exp lncRNA2× β lncRNA2 + Exp lncRNA3× β lncRNA3 + ••• + Exp lncRNAn × β lncRNAn.

### Analysis of risk signature

Patients were divided into high- and low-risk groups based on median values of risk score to determine the prognosis of signature. We used the survival package to calculate overall survival (OS) and progression-free survival (PFS) for patients with LUAD in various groups and performed univariate and multivariate independent prognostic analyses to evaluate the independent prognostic value of the risk prediction signature. The pheatmap package was used to plot patient survival status and lncRNAs expression heatmap based on the risk scores. The survivalROC package was used to calculate the 1-, 3-, and 5-year area under the ROC curve (AUC) of signature in the training, testing, and all groups.

### Construction of nomogram and validation of clinical subgroups

Nomograms were constructed for age, gender, T stage, N stage, TNM stage, and risk score using the survival and rms packages. Calibration curves were plotted to show the difference between the predicted and actual outcomes of the nomogram. C-index curves are used to verify the accuracy of the signature in predicting the survival of patients with LUAD. Finally, patients were divided into stages I–II and stages III–IV to determine if the signature can be used for patients with LUAD at different stages.

### Principal Component Analysis (PCA) and functional enrichment analysis

PCA analysis was performed using the limma and scatterplot3d packages to explore the distribution of patients with different risk scores. The gene ontology (GO) and Kyoto Encyclopedia of Genes and Genomes (KEGG) enrichment analysis of cuproptosis-related lncRNAs was performed using the clusterProfiler package, and *P* value < 0.05 and false discovery rate (FDR) < 0.05 were considered statistically significant.

### Immune-related functional analysis and tumor mutation burden (TMB) analysis

The limma GSVA package was used to analyze differences in immune-related functions in patients with LUAD, and *P* < 0.05 was considered statistically significant. The pheatmap package was used to visualize the results, and the maftools package was used to compare the relationship between risks core and TMB. We used the survival package to determine the difference between TMB and patient survival. A *P* value of <0.05 was considered statistically significant.

### Immunotherapy analysis and pharmaceutical screening

We downloaded tumor immune dysfunction and exclusion (TIDE) data for non-small cell lung cancer (NSCLC) from http://tide.dfci.harvard.edu/. The TIDE algorithm is a new development that uses TIDE-scores to accurately predict the efficacy of immunotherapy drugs received by patients [23]. Jiang et al. found that TIDE scores have been shown to represent the efficacy of immune drugs (anti-PD1, anti-CTLA4) in melanoma patients, with higher TIDE scores being associated with better outcomes [23]. Using the ggpubr and limma packages, we analyzed the relationship between risk score and TMB, with *P* < 0.05 considered statistically significant. Screening of therapeutic agents and observation of drug sensitivity using the pRRophetic, ggplot2, and ggpubr packages with pFilter = 0.001 and corPvalue = 0.001.

### Validation the prognostic of cuproptosis-related lncRNAs in GEO datasets

Patients were divided into high and low groups based on the expression of cuproptosis-related lncRNAs. We used the survival package for survival analysis of cuproptosis-related lncRNAs in GSE30219, GSE31210, and GSE37745 datasets, with *P* < 0.05 considered statistically significant.

## Results

### Identification of cuproptosis-related lncRNAs and construction of a prognostic signature

**Fig. 1:**
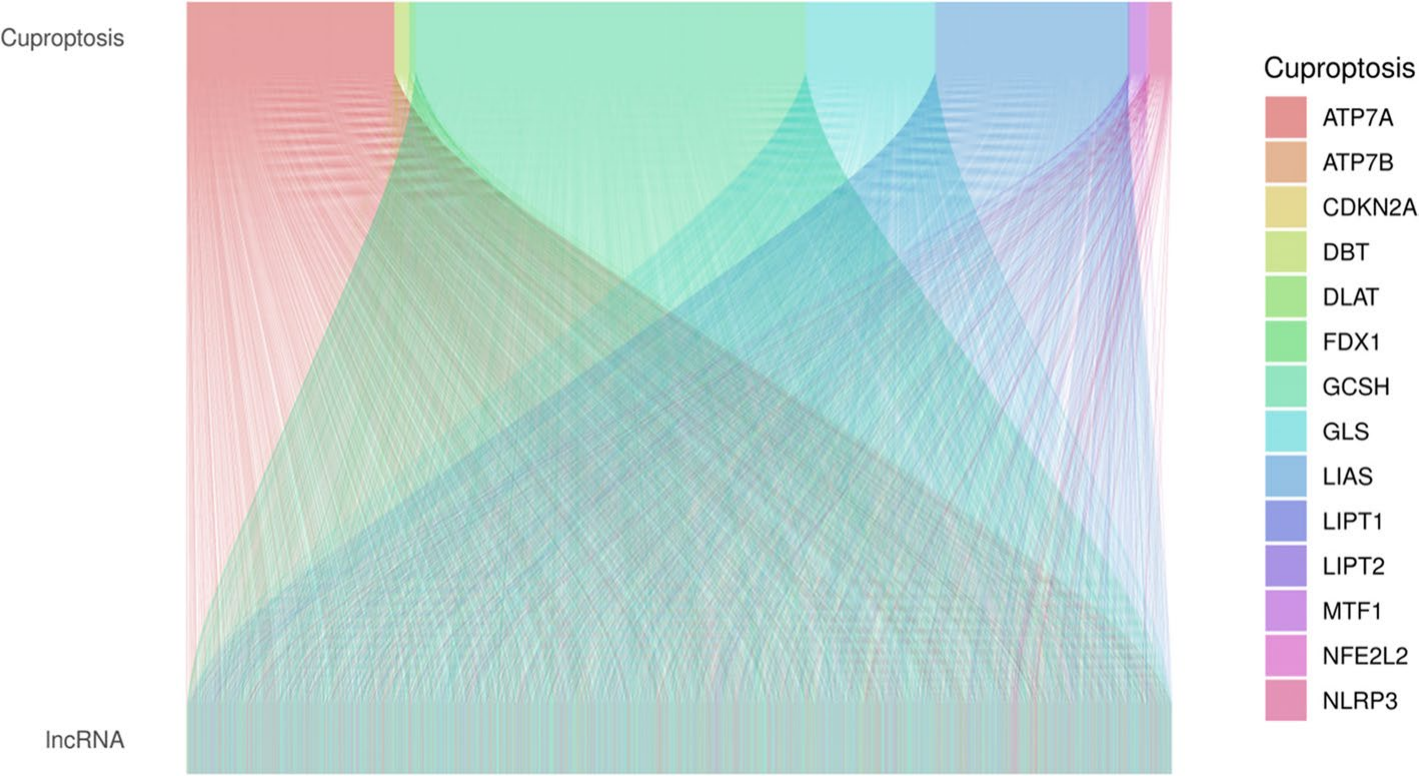
Sankey diagram showed the results of cuproptosis-related genes and cuproptosis-related lncRNA co-expression

**Fig. 2:**
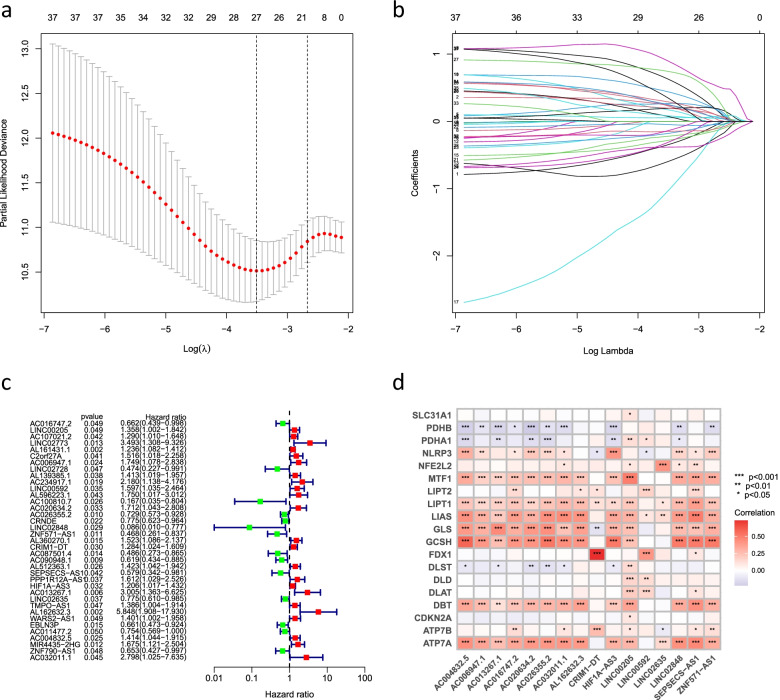
Identification of the cuproptosis-related lncRNAs. **a** LASSO regression screened of cuproptosis-related lncRNAs at the minimum point of cross-validation. **b** Trajectory of each independent variable. **c** Forest plot showed different lncRNAs for high and low risk, with red representing high-risk lncRNAs and green representing low-risk lncRNAs. **d** Correlation heatmap showed the relationship between cuproptosis-related lncRNAs and cuproptosis-related genes for the signature. Red represents positive correlations and blue represents negative correlations

With the |R| >0.4 and *P* < 0.001 as the analysis criteria, 2244 cuproptosis-related lncRNAs were identified from 16,876 lncRNAs and 19 cuproptosis-related genes. The co-expression relationships between the cuproptosis-related genes and cuproptosis-related lncRNAs were visualized using a Sankey diagram (Fig. 1). In the training group, Lasso Cox regression analysis identified cuproptosis-related lncRNAs, univariate Cox regression analysis identified 37 lncRNAs, and multivariate COX analysis identified 16 lncRNAs as independent prognostic factors. The risk score of each sample was then calculated based on the expression of 16 lncRNAs (Fig. 2a–c). Risk score =(−0.79670054952258*AC016747.2)+(0.454977942296554*LINC00205)+(0.971038537693743*AC006947.1)+(0.466282143262505*LINC00592)+(0.580816870667386*AC020634.2)+(−0.364069056069612*AC026355.2)+(−2.94320726773469*LINC02848)+(−0.760200155891508*ZNF571-AS1)+(0.429955081610087*CRIM1-DT)+(−1.0655839243752*SEPSECS-AS1)+(0.272457956361295*HIF1A-AS3)+(1.56215847973464*AC013267.1)+(−0.364308918322871*LINC02635)+(1.84725474576194*AL162632.3)+(0.646354490290833*AC004832.5)+(1.01912906171161*AC032011.1). The correlation heatmap also showed the relationship between the cuproptosis-related genes and lncRNAs (Fig. 2d).

### Survival analysis of the signature

**Fig. 3:**
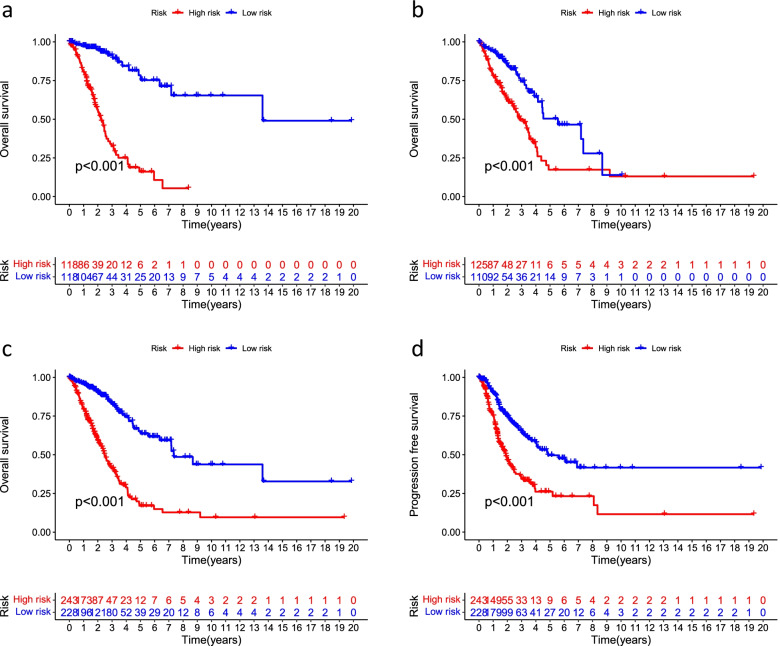
Kaplan–Meier survival analyses of patients. Patients were divided into high-risk and low-risk group based on the median risk score to predict overall survival (OS) and progression-free survival (PFS) in each subgroup. **a** OS in the training group. **b** OS in the testing group. **c** OS in all groups. **d** PFS in all groups

**Fig. 4:**
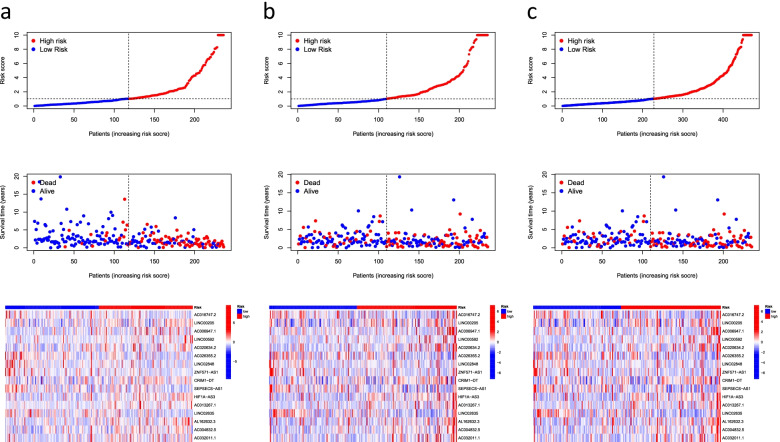
Predicting the performance of characteristics. Risk curves represented the distributed survival status of LUAD patients with different risk scores; heatmap represented the characteristics of lncRNAs in the **a** training group, **b** testing group, and **c** all groups

For a better assessment of the prognostic value of the risk signature based on the median value of the risk score as the cutoff, the patients were divided into high- and low-risk groups. We found that OS and PFS were significantly shorter in the high-risk group than in the low-risk group in the training, testing, and all groups (Fig. 3). The risk curves reflect the relationship between the risk score and survival status of patients with LUAD, and we found that mortality was higher in the high-risk patients than the low-risk patients. The heatmap showed high- and low-risk levels for 16 lncRNAs. For example, LINC00205, LINC00592, and AL162632.3S were high-risk lncRNAs, whereas AC026355.2, LINC02848, and ZNF571-AS1 were low-risk lncRNAs (Fig. 4).

### Independent analysis of prognostic factors

**Fig. 5:**
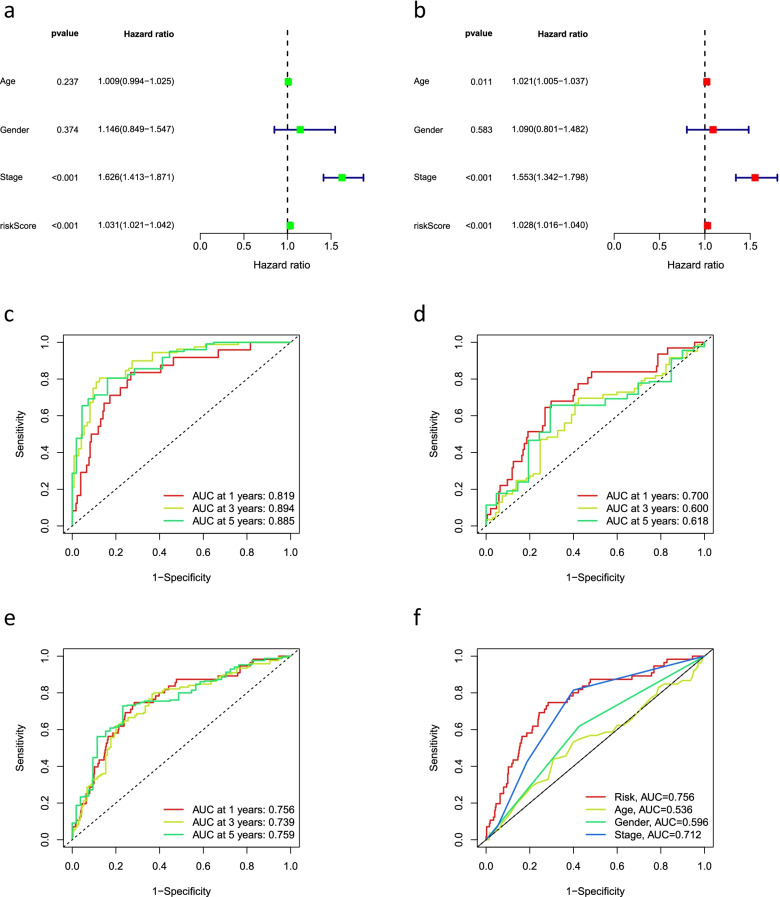
The prognostic value of the signature for LUAD. **a** Univariate and **b** multivariate independent prognostic analysis to analyze that the risk score was independently associated with OS. 1-, 3-, and 5-year area under the ROC curve (AUC) of signature in the **c** training, **d** testing, and **e** all groups. **f** ROC curves for the riskscore (AUC=0.756) and other clinical features

Univariate and multivariate Cox regression analyses were performed to determine whether the risk signature had the potential to be a prognostic factor independent of other clinical characteristics. Multivariate Cox regression results showed that stage (hazard ratio [HR] = 1.553, 1.342–1.798; *P* < 0.05) and risk score (HR = 1.028, 1.016–1.040; *P* < 0.05) were independently associated with OS, indicating that the risk signature is an independent prognostic factor for patients with LUAD (Fig. 5a, b). Further, we used ROC curves to assess the predictive accuracy of the risk score. The AUC for the risk score was 0.756, which was better than those for age (0.536), gender (0.596), and stage (0.712) (Fig. 5f). Similarly, in the all group, the AUCs for 1-, 3-, and 5-year OS were 0.756, 0.739, and 0.759, respectively (Fig. 5e), suggesting that the signature has reliable diagnostic significance. The ROC curves also showed that the signature could predict OS in the training and testing groups (Fig. 5c, d).

### Construction of a predictive nomogram and PCA

**Fig. 6:**
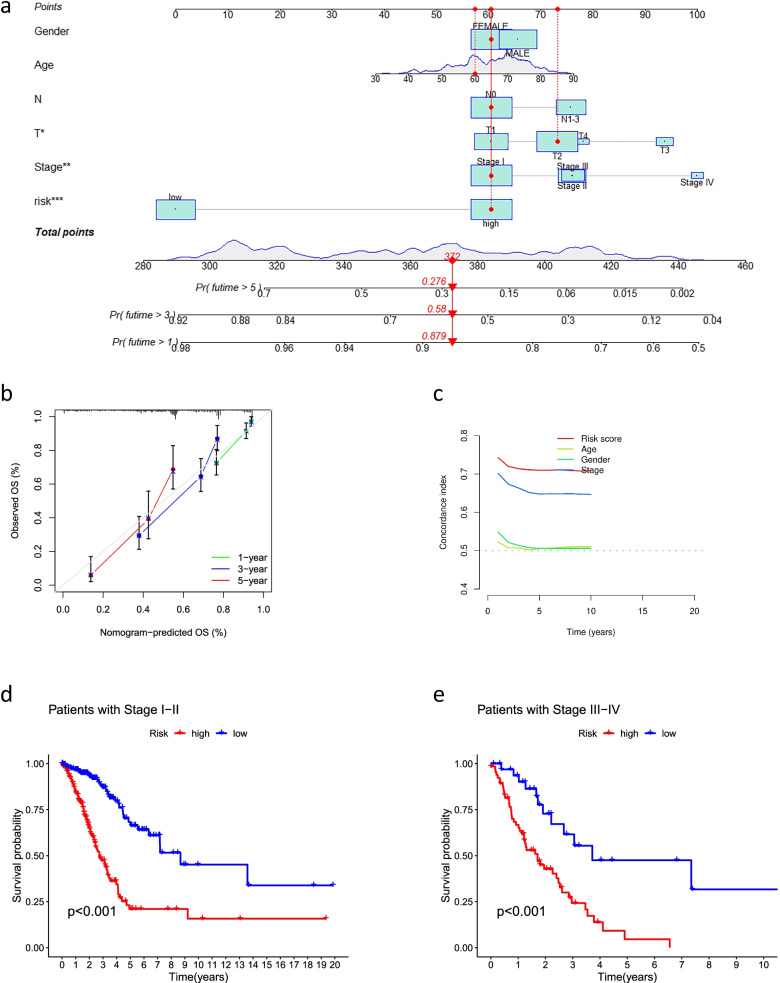
Nomogram and clinical subgroups for predicting LUAD outcomes. **a** Prognostic nomogram to predict the OS in LUAD. **b** Calibration curves for 1, 3, and 5 years. **c** C-Index curve analyzed the concordance index of the risk score. Patients were grouped to see if the model was applicable to LUAD patients at **d** stages I–II and **e** stages III–IV

**Fig. 7:**
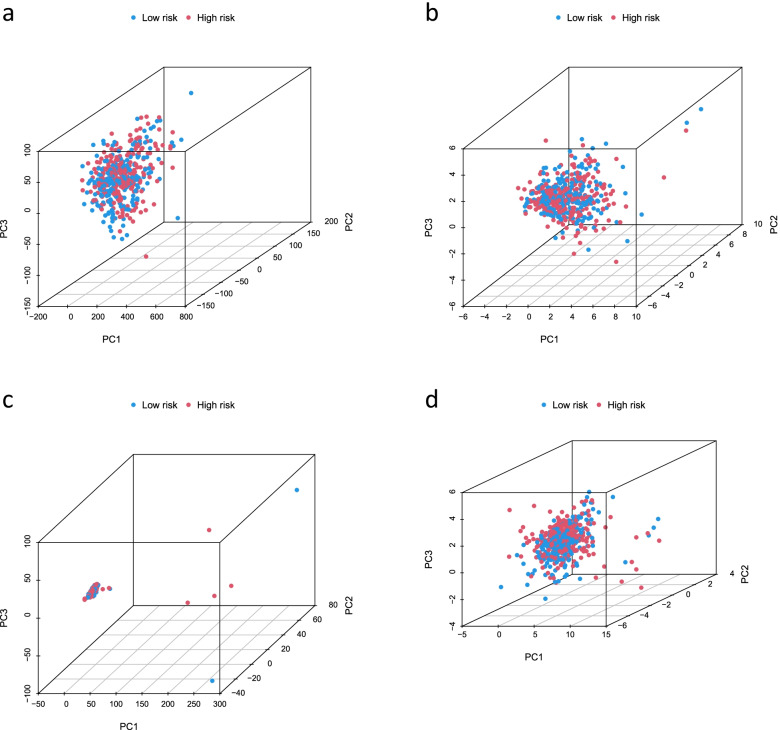
PCA analysis. PCA analysis observed the distribution of patients according to **a** all genes. **b** Genes associated with cupulocytosis. **c** lncRNAs associated with cupulosis. **d** Risk lncRNAs

We constructed a nomogram using factors such as age, gender, TNM stage, T stage, risk score, and N stage from the signature (the M stage showed several uncertain values, which were not included in this study). The nomogram could reliably predict the 1-, 3-, and 5-year OS of patients with LUAD (Fig. 6a, b). Furthermore, we found that the C-index values of the risk score were higher than those of other clinical characteristics, such as age, gender, and stage (Fig. 6c). We further analyzed the significant differences (*P* < 0.05) in OS between the high-risk and low-risk groups of patients at different stages (stages I–II and III–IV) (Fig. 6d, e), which suggested that the signature has high predictive accuracy and can be used to compare the survival of patients at different stages. Finally, we performed PCA to observe the distribution of patients for all genes, cuproptosis-related genes, cuproptosis-related lncRNAs, and risk lncRNAs, and the results showed a clear distribution, indicating that these lncRNAs can be reliably used to construct the signature (Fig. 7).

### Functional enrichment analysis and immune-related functional analysis

**Fig. 8:**
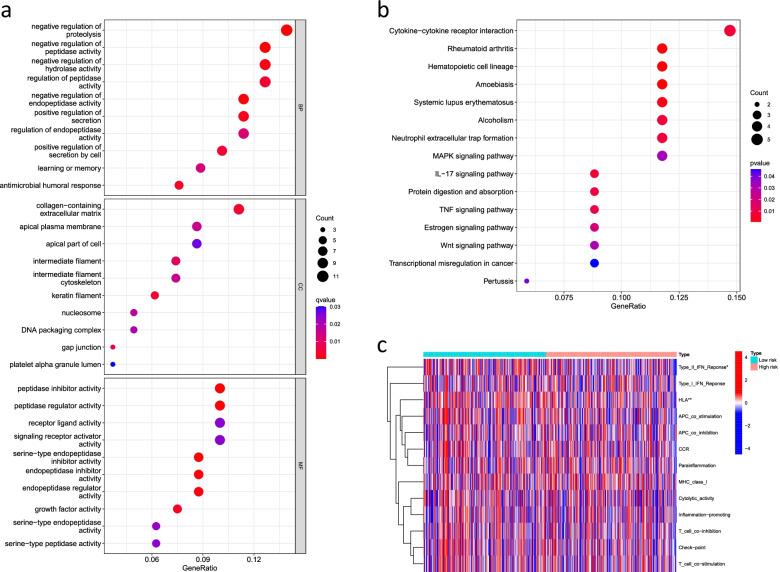
Functional enrichment analysis and immune-related functional analysis. **a** GO enrichment analyses of the 16 cuproptosis-related lncRNAs. **b** KEGG enrichment analyses of the 16 cuproptosis-related lncRNAs. **c** Immune-related functions of the 16 cuproptosis-related lncRNAs

GO analysis results showed that the cuproptosis-related lncRNAs enriched in the negative regulation of proteolysis, regulation of peptidase activity, and negative regulation of hydrolase activity (Fig. 8a). KEGG analysis showed that these cuproptosis-related lncRNAs may be related to the cytokine−cytokine receptor interaction, neutrophil extracellular trap formation, and mitogen-activated protein kinase signaling pathways, suggesting that these lncRNAs are involved in tumor development (Fig. 8b). Furthermore, we analyzed immune-related functions to evaluate the immune status of the low-risk and high-risk groups, and the results showed that the type III interferon (IFN) response was significantly more active in the low-risk group than in the high-risk group, but no significant differences were noted for other immune functions (*P* > 0.05) (Fig. 8c).

### TMB analysis and drug sensitivity analysis

**Fig. 9:**
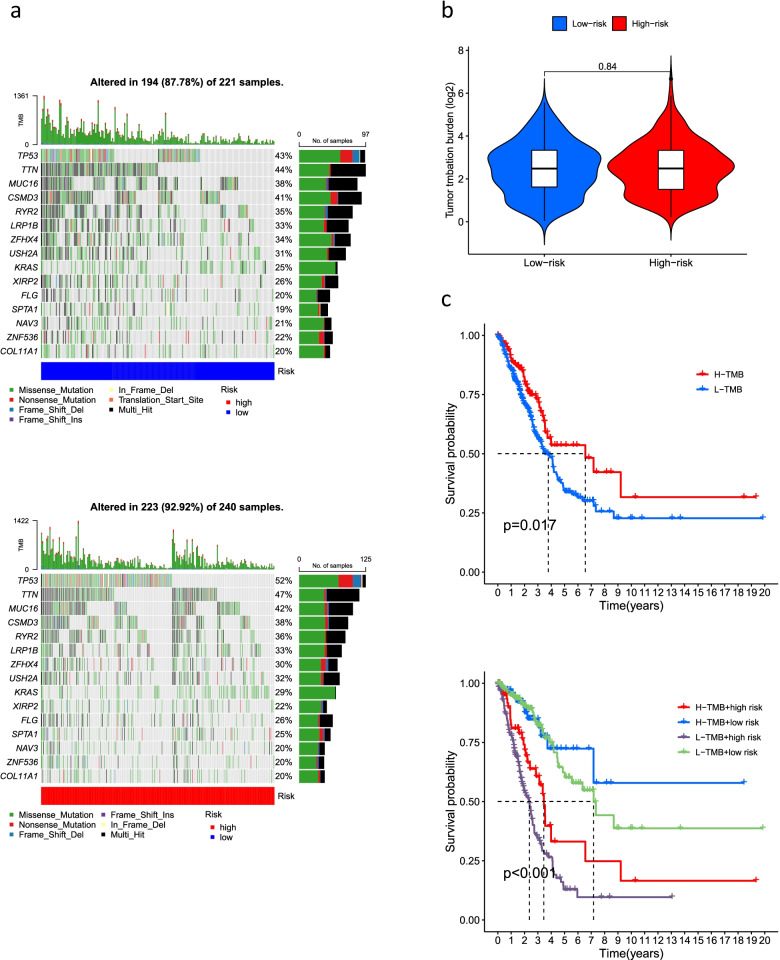
The relationship between TMB and the signature. **a** Waterfall plot revealed the top 15 mutation genes in LUAD for the low-risk (221 samples) and high-risk (240 samples) groups. **b** Differential TMB in high-risk and low-risk groups in LUAD. **c** Survival curves for the high-TMB and low-TMB groups in LUAD and a combined TMB-risk survival curve

**Fig. 10:**
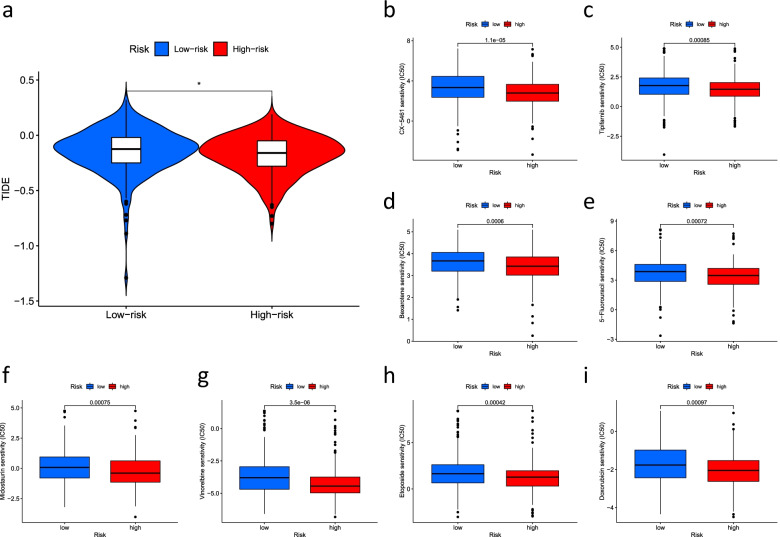
Immunotherapy and drug sensitivity. **a** Tumor immune dysfunction and exclusion (TIDE) algorithm analysis for high-risk and low-risk groups. *Represents statistically significant differences. Observed the drug sensitivity of **b** CX-5461, **c** Tipifarnib, **d** Bexarotene, **e** 5-Fluorouracil, **f** Midostaurin, **g** Vinorelbine, **h** Etoposide, and **i** Doxorubicin

We used the maftools algorithm to observe mutations in the high- and low-risk groups and showed that for most genes, the frequency of mutations was higher in the high-risk group than in the low-risk group (*TP53*: low risk, 43%; high risk 52%. *TTN*: low risk, 44%; high risk, 47%; *MUC16*: low risk, 38%; high risk, 42%) (Fig. 9a). Furthermore, the difference in TMB between the high-risk and low-risk groups was not significant (*P* = 0.84) (Fig. 9b). The reason behind this result needs to be further explored. We further investigated a probable difference in survival between patients with high and low TMB. As shown in Fig. 9c, OS was significantly better in the high TMB group than in the low TMB group (*P* < 0.05). In addition, the difference in sensitivity to immunotherapy between patients in the high- and low-risk groups was further investigated using the TIDE algorithm (http://tide.dfci.harvard.edu/). The TIDE algorithm is a recently developed tool for determining the efficacy of tumor immune checkpoint therapy [23]. In the present study, we found a higher TIDE score in the low-risk group than in the high-risk group (Fig. 10a). However, whether immunotherapy is better for low-risk patients with LUAD than high-risk patients with LUAD needs to be further explored. We used the pRRophetic packages to screen the potentially effective antitumor drugs, including masitinib, tipifarnib, bexarotene, 5-fluorouracil, midostaurin, vinorelbine, etoposide, and doxorubicin (Table 1). We further analyzed the sensitivity of these drugs and found that patients in the high-risk group had lower IC50 values (concentration that inhibits cell growth by 50%), representing a higher sensitivity of the drugs in patients at high risk (Fig. 10b–i).

**Table 1:** Anti-tumor drugs and indications

Anti-tumor drugs	Indications
Masitinib	Melanoma
Tipifarnib	Lung cancer, lymphoma, pancreatic cancer
Bexarotene	Cutaneous t cell lymphoma
5-Fluorouracil	Liver cancer, stomach cancer
Midostaurin	Lung cancer, hematology tumor
Vinorelbine	Non-small cell lung cancer, breast cancer, malignant lymphoma
Etoposide	Lung cancer, malignant lymphoma, malignant germ cell tumor
Doxorubicin	Cholangiocarcinoma

### Prognostic analysis of the GEO datasets

**Fig. 11:**
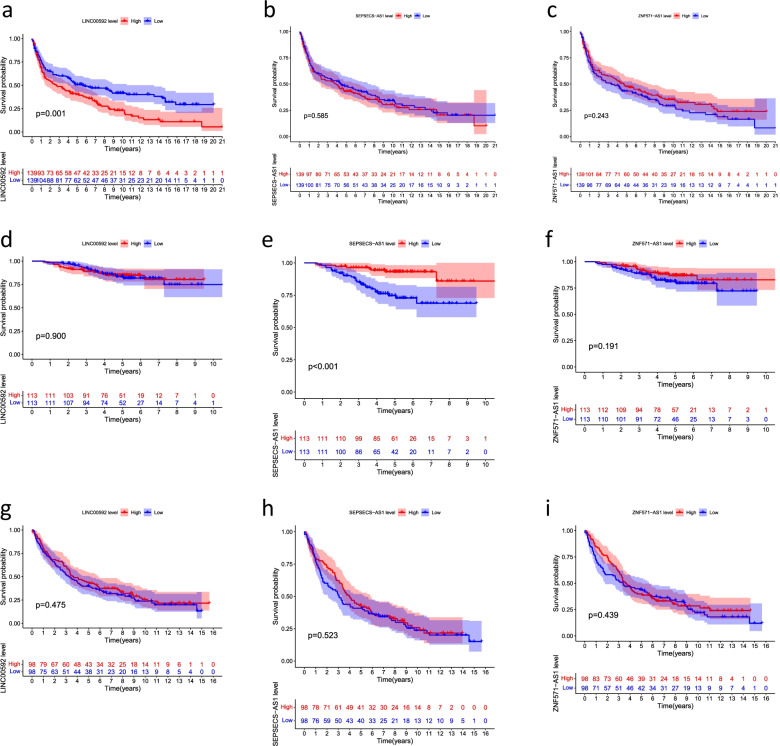
Prognostic validation of single cuproptosis-related lncRNAs in the GEO datasets. **a** LINC00592, **b** SEPSECS-AS1, and **c** ZNF571-AS1 in GSE30219. **d** LINC00592, **e** SEPSECS-AS1, and **f** ZNF571-AS1 in GSE31210. **g** LINC00592, **h** SEPSECS-AS1, and **i** ZNF571-AS1 in GSE37745

Among the 16 Cuproptosis-related lncRNAs (AC016747.2, LINC00205, AC006947.1, LINC00592, AC020634.2, AC026355.2, LINC02848, ZNF571-AS1, CRIM1-DT, SEPSECS-AS1, HIF1A-AS3, AC013267.1, LINC02635, AL162632.3, AC004832.5, AC032011.1) in GSE30219, GSE31210, GSE37745, only LINC00592, ZNF571-AS1, and SEPSECS-AS1 was observed, and the remaining 13 cuproptosis-related lncRNAs had not been observed. Then, we performed the survival analysis of single cuproptosis-related lncRNAs in GSE30219, GSE31210, and GSE37745 for LINC00592, ZNF571-AS1, and SEPSECS-AS1, respectively, and the results are shown in Fig. 11.

## Discussion

Lung cancer has the highest incidence and mortality rate among malignant tumors in China, and LUAD, a type of lung cancer, has a very poor prognosis [24]. Currently available lung cancer screening tools, such as low-dose computed tomography, are effective in reducing lung cancer mortality but have a high false-positive rate. Therefore, the construction of a reliable lung cancer risk signature to accurately determine the prognosis and survival of patients with LUAD is of great importance in the prevention and control of LUAD [25]. lncRNAs are a class of non-protein-coding RNAs that are >200 nt in length and account for more than 80% of lncRNAs [26]. lncRNAs play an important regulatory role in lung cancer. A study found that lncRNA KTN1-AS1 acts as a pro-oncogene in NSCLC and can affect the NSCLC cell cycle by regulating cyclin-dependent kinase (CDK) 1, suggesting that lncRNAs may act as a novel lung cancer biomarker and therapeutic target [27]. Cuproptosis is a unique form of cell death that has been identified recently [18–22]. This copper-dependent cell death occurs via the direct binding of copper to acylated lipid components of the TCA cycle in mitochondrial respiration, leading to the aggregation of acylated proteins and the subsequent decrease in iron-sulfur proteins, resulting in proteotoxicity and ultimately cell death [28]. Although clinical trials of the copper ionophore micromolecule anticancer drug elesclomol [29] have been conducted, their results have been unsatisfactory. However, the co-regulatory role of cuproptosis and lncRNAs in LUAD needs to be further investigated.

In the present study, cuproptosis-related lncRNAs were obtained by the co-expression of lncRNAs and cuproptosis-related genes. A total of 16 prognostic cuproptosis-related lncRNAs, including including AC016747.2, LINC00205, AC006947.1, LINC00592, AC020634.2, AC026355.2, LINC02848, ZNF571-AS1, CRIM1-DT, SEPSECS-AS1, HIF1A-AS3, AC013267.1, LINC02635, AL162632.3, AC004832.5, and AC032011.1 were obtained by univariate and multivariate Cox regression analyses, and the prognostic signature was constructed. The results of ROC, survival, nomogram, and pheatmap analyses showed that the prognostic features of the 16 cuproptosis-related lncRNAs were accurately distinguished between the high-risk and low-risk groups and early-stage and late-stage patients, reliably predicting outcomes in patients with LUAD, and these lncRNAs were prognostic factors independent of other common clinical characteristics. Of the 16 cuproptosis-related lncRNAs in the LUAD signature, only LINC00205 has been shown to function in cancer. LINC00205 was an overexpressed oncogene involved in tumor progression in lung, liver, gastric cancers, and retinoblastoma [30–35]. In hepatocellular carcinoma, LINC00592 was associated with patient prognosis and could target the cold shock domain-containing E1 protein, CDK6, miR-122-5p, and epoxide hydrolase 1, promoting the proliferation of hepatocellular carcinoma cells [30–33]. Li et al. found that LINC00205 targets the oncogene miR-185-5p as a potential therapeutic target in lung and gastric cancers. LINC00205 promoted the proliferation and migration of gastric cancer cells by inhibiting miR-26a [36]. Zhang et al. suggested that LINC00205 targeting the high mobility group box 1 protein promoted the proliferation of neuroblastoma, which was correlated with OS [34]. The roles of other 15 lncRNAs were first investigated in cancers. Further, the GO and KEGG pathway analyses revealed that cuproptosis-related lncRNAs may be associated with LUAD development.

We then analyzed the relationship between immune-related function, TMB, and risk scores in patients with LUAD and found that type III-IFN response was inhibited in high-risk patients. IFNs interfere with virus replication in vitro, and type III-IFNs are an essential component of antiviral immunity [37]. Type III-IFN-response inhibition can be one of the main causes of immune escape, and its activation is essential to maintain immune potency. TMB is often used as a predictive biomarker for an immune checkpoint blockade in melanoma, lung, and bladder cancers [38–40]. We found a significant decrease in the survival of patients with high TMB (*P* < 0.05) and an increase in *TP53* and *TTN* expression in patients at high risk. *TP53* is a frequently mutated oncogene in human cancers, affecting the development of breast, lung, bladder, esophageal, prostate, pancreatic, and colorectal cancers and is involved in the normal physiology and metabolism of diabetes, liver, and cardiovascular diseases [40–44]. *TP53* is also associated with the survival of patients with multiple cancers [45]. *TTN* participates in the development of multiple cancers. Shen et al. found that *TTN* can target the miR-376a-3p/PUM2 axis and promote the growth of endometrial cancer cells, suggesting that *TTN* may be a therapeutic target for endometrial cancer [46]. Fu et al. found that *TTN* acts as a pro-oncogene in osteosarcoma by targeting miR-134-5p and promoting the expression of the malignant brain tumor domain-containing 1 gene, which ultimately promotes the growth of osteosarcoma cells [47]. Xiao et al. found that in bladder cancer, downregulated *TTN* expression inhibited the proliferative capacity of breast cancer cells [47]. Our results are consistent with those of previous studies [40–48], suggesting that *TP53* and *TTN* can act as targets for cancer immunotherapy. In the present study, we found that the TIDE score in the low-risk group was higher than that in the high-risk group; however, the difference in the effect of immunotherapy on high-risk patients with LUAD and low-risk patients with LUAD still needs to be further explored. PD-L1/PD-1, CD24/Siglec-10, epithelial-mesenchymal transition signaling, hypoxia-/hypoxia-inducible factor 1α drivers, and CCAAT/enhancer-binding protein β transcription factors affect immunotherapy, a recently prevalent oncology treatment [49–53]. Notably, the role of ncRNAs in immunotherapy has been widely researched. Zhou et al. found that miR-1468-5p could suppress the immune system by targeting lymphatic PD-L1, causing cervical cancer cells to evade treatment [54]. In neuroblastoma, the oncogenic factor miR-186 attenuates the efficacy of immunotherapy by inhibiting natural killer cells [55]. Zhang et al. identified lncRNA GATA3-AS1 as a potential therapeutic target for breast cancer, promoting immune escape by targeting GATA3 [56]. However, the relationship between lncRNAs and immune-related functions is not fully understood, and in the future, we will evaluate the prognosis of patients with lung cancer regarding immune cell infiltration and explore the role of immune cells in targeted therapy for patients with LUAD. We then used the pRRophetic algorithm to screen for effective drugs for tumor immunotherapy and explored the sensitivity of these drugs, which have been used in lung, lymphoma, pancreatic, breast, kidney, and bile duct cancers, followed by the observation that patients at high risk are more sensitive to anticancer drugs. However, the drug mechanisms and their impact on LUAD progression need further study. Despite the prognostic value of the signature, this study still has some limitations. First, the prognostic results of cuproptosis-related lncRNAs in the GEO dataset were less than favorable. Second, our findings are based on public databases and lack available information on clinical specimens. Moreover, we will continue to focus on and improve this limitation in the future.

## Conclusion

In the present study, we constructed a cuproptosis-related lncRNA signature for LUAD and analyzed the relationship between risk score-based groups and TMB, immunotherapy, and drug sensitivity. This study provides new insights into the prediction of survival of patients with LUAD and the efficacy of clinical treatment.

## Data Availability

Data supporting the findings of this study are available from the respective authors upon reasonable request.
